# Designing customized temporomandibular fossa prosthesis based on envelope surface of condyle movement: validation via *in silico* musculoskeletal simulation

**DOI:** 10.3389/fbioe.2023.1273263

**Published:** 2023-10-31

**Authors:** Jun-Lin Wang, Jing Wang, Ke-Nan Chen, Jian-Qiao Guo, Xiang-Liang Xu, Chuan-Bin Guo

**Affiliations:** ^1^ Department of Oral and Maxillofacial Surgery, Peking University School and Hospital of Stomatology, National Center of Stomatology, National Clinical Research Center for Oral Diseases, National Engineering Research Center of Oral Biomaterials and Digital Medical Devices, Beijing Key Laboratory of Digital Stomatology, Research Center of Engineering and Technology for Computerized Dentistry, Ministry of Health, NMPA Key Laboratory for Dental Materials, Beijing, China; ^2^ MOE Key Laboratory of Dynamics and Control of Flight Vehicle, School of Aerospace Engineering, Beijing Institute of Technology, Beijing, China

**Keywords:** articular fossa prosthesis, envelope surface, musculoskeletal simulation, flexible multibody dynamics, temporomandibular joint, mandibular movement

## Abstract

**Objective:** This study presents an innovative articular fossa prosthesis generated by the envelope surface of condyle movement, and compares its mandible movements, muscle activities, and joint reaction forces with two temporomandibular joint (TMJ) prostheses using multibody musculoskeletal simulation.

**Methods:** A healthy 23-year-old female was recruited for this study. Cone-beam computed tomographic (CBCT) was performed to reconstruct the mandibular bone geometry. A customized TMJ fossa prosthesis was designed based on the subject-specific envelope surface of condyle movement (ESCM). Mandibular kinematics and jaw-closing muscle electromyography (EMG) were simultaneously recorded during maximum jaw opening-closing movements. To validate our prosthesis design, a mandibular musculoskeletal model was established using flexible multibody dynamics and the obtained kinematics and EMG data. The Biomet fossa prosthesis and the ellipsoidal fossa prosthesis designed by imitating the lower limb prostheses were used for comparison. Simulations were performed to analyze the effects of different fossa prostheses on jaw opening-closing motions, mandibular muscle activation, and contact forces.

**Results:** The maximum opening displacement for the envelope-based fossa prosthesis was greater than those for Biomet and ellipsoidal prostheses (36 mm, 35 mm, and 33 mm, respectively). The mandibular musculoskeletal model with ellipsoidal prosthesis led to dislocation near maximal jaw opening. Compared to Biomet, the envelope-based fossa reduced the digastric and lateral pterygoid activation at maximal jaw opening. It also reduced the maximal resistance to condylar sliding on the intact side by 63.2 N.

**Conclusion:** A customized TMJ fossa prosthesis was successfully developed using the ESCM concept. Our study of musculoskeletal multibody modeling has highlighted its advantages and potential. The artificial fossa design successfully achieved a wider condylar range of motion. It also reduced the activation of jaw opening muscles on the affected side and resistance on the intact side. This study showed that an ESCM-based approach may be useful for optimizing TMJ fossa prostheses design.

## 1 Introduction

The temporomandibular joint (TMJ) is the only movable joint of the human oral and maxillofacial region, and is actively involved in several daily activities (Zheng et al., 2019). TMJ disorders, such as tumors and ankylosis, can affect its integrity and cause joint dysfunction (Mercuri, 2000). Total joint replacement is an effective method for TMJ reconstruction and functional restoration (Sidebottom, 2008).

Currently, there are two commercially-available TMJ replacement systems approved by the Food and Drug Administration, Biomet Microfixation (Jacksonville, FL, USA) (Imola and Liddell, 2016) and TMJ Concepts (Ventura, CA, USA) (Wolford et al., 1994). Both these systems consist of condylar and fossa components (Kiehn et al., 1974). The TMJ Concepts prostheses are constructed using patient-specific cone-beam computed tomography (CBCT) data (Wolford et al., 2003). Meanwhile, Biomet has three stock components with different lengths and styles (Imola and Liddell, 2016). The Biomet artificial fossa is a flat and ellipsoidal surface, and the TMJ fossa and mandibular ramus may need to be trimmed in order to fit with it.

Existing TMJ prostheses were designed solely based on medical imaging, and cannot completely restore the physiological condylar kinematics (Zou et al., 2020). The natural condyle has sliding and rotational movements (van Loon et al., 1999). Although TMJ replacement significantly improves mandibular movement, the condylar kinematics for the prosthesis are different compared to the natural TMJ (Westermark, 2010; Gruber et al., 2015; Gonzalez-Perez et al., 2016). In particular, condylar sliding may be completely lost after TMJ replacement (Sonnenburg and Sonnenburg, 1985; Merlini and Palla, 1988; Mercuri et al., 1995). This may be because the geometry of the TMJ prosthesis restricts the condylar range of motion (ROM) (van Loon et al., 1999). *In vitro* experiments performed by Celebi et al.(2011) demonstrated that the artificial condyle is more deeply enclosed within the articular fossa compared to the natural condyle. This makes achieving the normal condylar ROM nearly impossible.

A function-based prosthesis may be designed by imitating the artificial joints of the lower limb. For example, the instantaneous center of rotation of the knee joint was considered when designing artificial knee implants (Walker, 2001). It has been observed that the physiological knee kinematics cannot be achieved by restoring its anatomical morphology alone (Wang et al., 2021). Similarly, TMJ fossa prostheses may also be custom-made based on the three-dimensional (3D) condylar movement. According to the finite element analysis using a canine model, this may result in a reasonable strain distribution (Xu et al., 2017). Reconstruction of the functional condylar surface, i.e., the envelope surface of condylar movement (ESCM), in normal adults was proposed by Huang et al. (2021). The use of the ESCM concept for designing TMJ fossa prostheses can allow physiologically accurate kinematics (Chen et al., 2022a; Chen et al., 2022b).

The ESCM surface concept has not yet been applied for the real-world design of human TMJ fossa prostheses. An important reason is the lack of systematic comparison of the effect of different TMJ prostheses on mandibular biomechanics. Radiographic, ultrasonic, magnetic, and optoelectronic tracking methods have previously been used for *in vivo* quantification of the mandibular kinematics (Woodford et al., 2020). Based on these measured kinematics data, multibody dynamics modeling provided an *in silico* method to investigate the hidden biomechanics of the mandibular musculoskeletal system. This has proven to be effective and reliable in quantifying the functional outcomes after mandibular surgery and reconstruction (Hannam et al., 2010; Hannam, 2011). Previous studies have also validated the feasibility of simulating maximal jaw opening-closing movements based on flexible multibody dynamics (Broser et al., 2021; Guo et al., 2022).

This study is the first to propose a TMJ fossa prosthesis design based on the ESCM concept. Functional outcomes of this fossa design, including mandibular movements, muscle activity, and joint reaction forces, were predicted based on musculoskeletal multibody simulations, and compared with those of the Biomet and ellipsoidal fossa prostheses. We hypothesized that the customized envelope-based fossa prosthesis would improve the functional outcomes, including condylar ROM and jaw opening muscle activations.

## 2 Materials and methods

### 2.1 Subject

This study was approved by the Institutional Review Board of Peking University School and Hospital of Stomatology, Beijing, China (Pkussirb-201947091). A 23-year-old female volunteer with no symptoms and signs of TMJ disorder or a history of TMJ disorder or orthodontic treatment was selected. Written informed consent to publish the findings was obtained.

### 2.2 CBCT

Skull base and mandibular CBCT scans (NewTom VG, NewTom, Imola, Italy; Voxel size: 0.3 mm, Field of view: 16 cm × 16 cm) were performed in the intercuspal position. The segmentation and 3D reconstructions were performed in stereolithographic format using CBCT data in the Proplan CMF software (version 3.0, Materialise, Leuven, Belgium).

### 2.3 Mandibular movements and electromyography (EMG)

The subject was instructed to perform two warm-up cycles and one test cycle of maximal opening-closing movements, beginning and ending in the maximum intercuspal position (Baqaien et al., 2007; Koeppel et al., 2015). The WINJAW ultrasound system (Zebris Medical GmbH, Isny, Germany) was used to record the mandibular motion. Mandibular position relative to the upper dentition was recorded using the Trios intraoral scanner (3Shape, Copenhagen, Denmark).

The WINJAW EMG device (Zebris Medical GmbH, Isny, Germany) was used to simultaneously record bilateral stomatognathic muscle activities during each cycle of mandibular movements. The electrodes were positioned on the anterior temporalis and masseter muscle bellies bilaterally. The subject was instructed to perform maximum voluntary contractions (MVCs) thrice to obtain the maximal muscle force-generating capacity for each muscle (Ferrario et al., 2004; Owashi et al., 2017). Raw EMG signals were rectified and low-pass filtered (Lloyd and Besier, 2003; Guo et al., 2020), and the data were normalized using the MVC values (Quental et al., 2012). The obtained dimensionless signal was used as input to analyze muscular activation based on the first-order activation dynamics equation (Thelen, 2003; Guo et al., 2022).

### 2.4 TMJ fossa protheses and ellipsoidal condyle design

**FIGURE 1:**
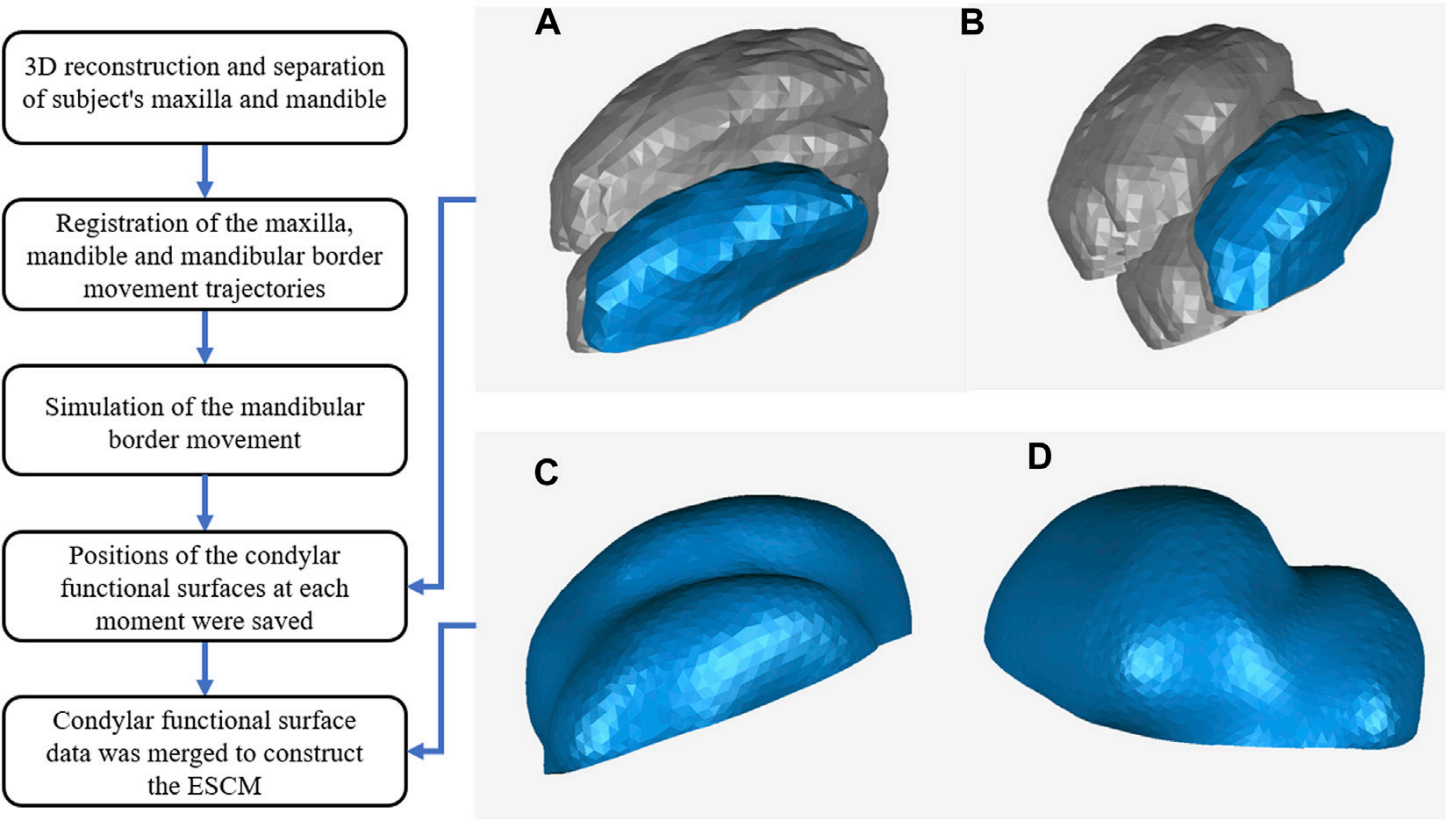
Procedure of obtaining the ESCM. The coronal section **(A)** and sagittal section **(B)** of positions of the condylar functional surfaces at each moment. The coronal section **(C)** and sagittal section **(D)** of ESCM generated by merging the condylar functional surface data.

**FIGURE 2:**
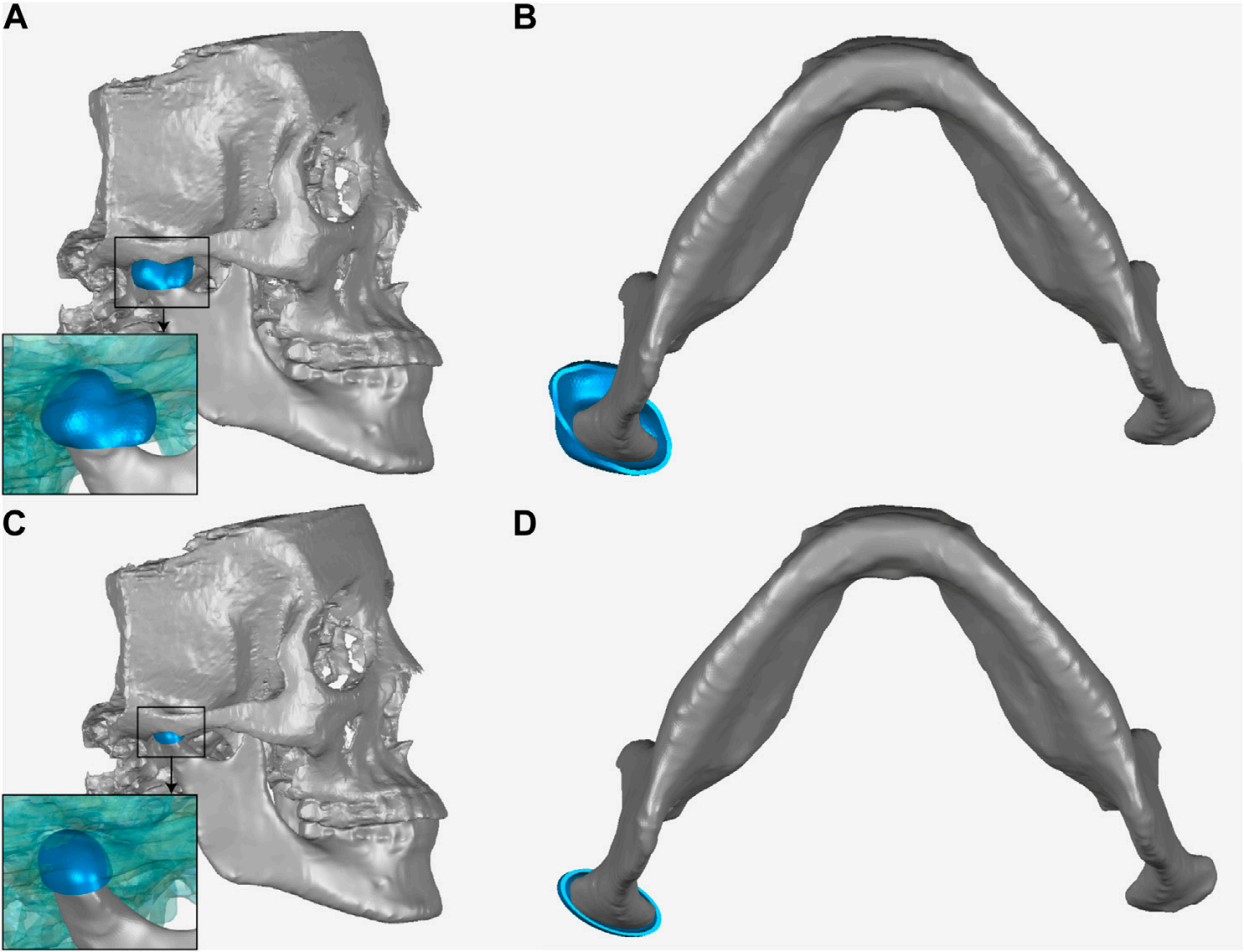
The sagittal section **(A)** and cross section **(B)** of envelope-based fossa prosthesis. The sagittal section **(C)** and cross section **(D)** of the ellipsoidal fossa prosthesis.

The TMJ fossa prothesis, based on the ESCM concept, was designed as follows. The maxilla, mandible and mandibular border movement trajectories were registered. Then according to the trajectories, the mandibular border movement was simulated, and the positions of the functional surfaces of the condyle at each moment were saved in the same 3D coordinated system. The condylar functional surface was defined as the coverage of the transverse ridge 6 mm forward. Condylar functional surface data was merged to construct the ESCM, as reported previously (Huang et al., 2021). The procedure used to obtain the ESCM is shown in Figure 1. Subsequently, the geometry of envelope surface was smoothened and refined using the Geomagic Studio software (version 2012, 3D Systems, Rock Hill, SC, USA). The envelope surface was uniformly thickened by 1 mm, and the customized fossa prosthesis was obtained (Figures 2A, B).

The Biomet fossa used in this study was obtained by increasing the overall size of the stock fossa prosthesis by 25% to fit the shape of the subject’s natural fossa. An ellipsoidal fossa prosthesis was obtained by setting a hemi-ellipsoid to fit the subject’s natural condyle (Figures 2C, D). The ellipsoidal fossa prosthesis was designed by imitating the lower limb joint implants and ignoring the condylar translation. The ellipsoidal fossa prosthesis was used to demonstrate the importance of condylar movements in TMJ prosthesis designing. Previous studies (Oberg et al., 1971; Zhao et al., 2019) have reported that the mandibular condyles of most adults are nearly ellipsoidal. The major (mediolateral) axis of the condyle is twice as long as the minor (anteroposterior) axis. Similarly, the mediolateral to anteroposterior diameter ratio of the ellipsoidal fossa prosthesis was 2:1.

**FIGURE 3:**
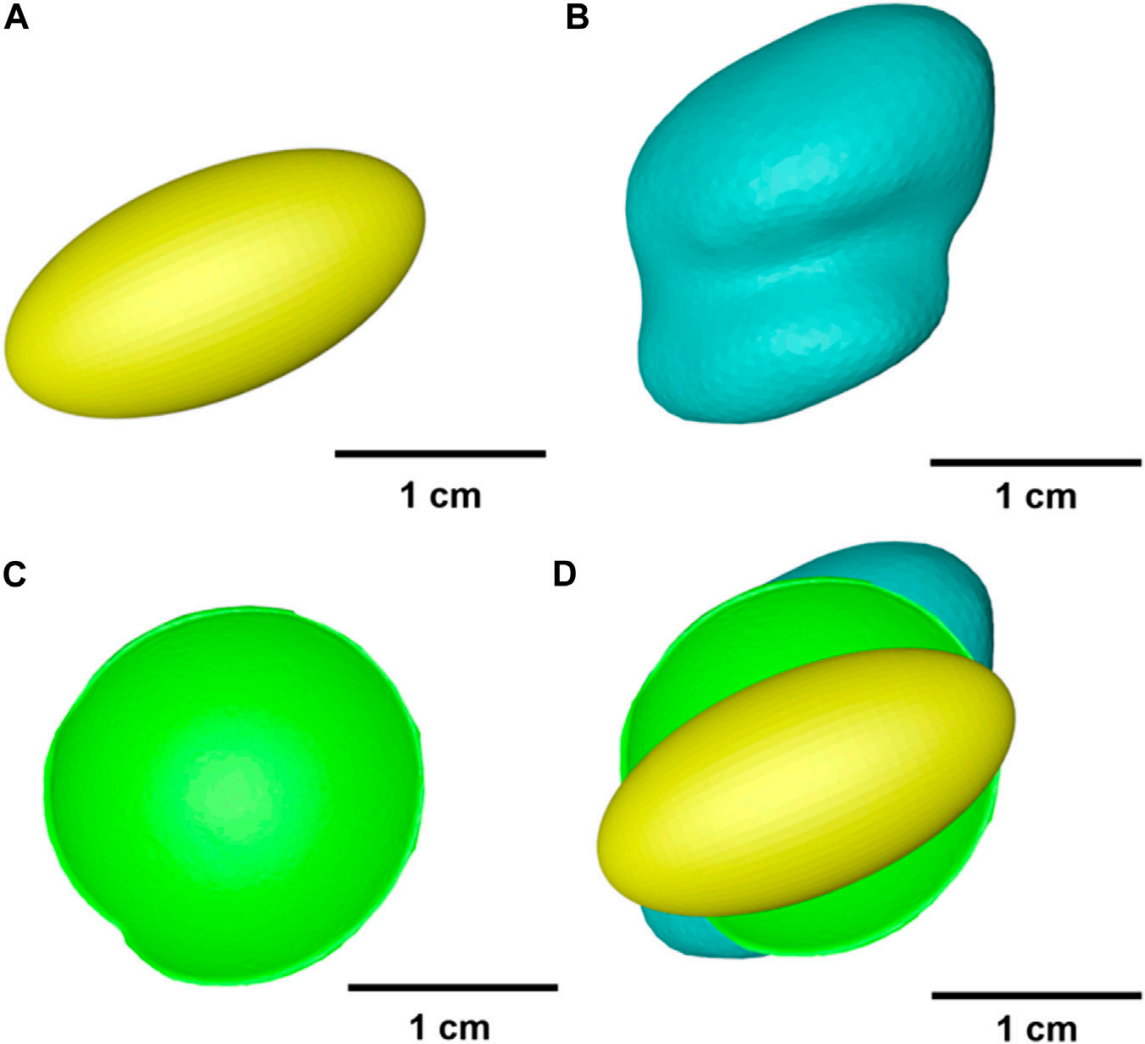
Geometry of different fossa prostheses. **(A)** Ellipsoidal fossa prosthesis. **(B)** Envelope-based fossa prothesis. **(C)** Biomet fossa prosthesis. **(D)** Comparison of the range of condylar motion.

The condylar ROMs for different fossa prostheses are shown in Figure 3. A least-squares fit of the subject-specific CBCT was used to determine the condylar radius, assuming the condylar surface to be frictionless (Yao et al., 2011; Modenese and Kohout, 2020; Guo et al., 2022). The condyle on the intact side was kept in the natural shape.

### 2.5 Mandibular multibody musculoskeletal model

Mandibular subject-specific musculoskeletal modeling and simulation were based on a study by Guo et al. (2022). The subject-specific mandibular and skull geometry was obtained from the reconstructed CBCT data (Koolstra and van Eijden, 2005). The mandibular model was driven by 24 muscle bundles (de Zee et al., 2007), which were discretized by a flexible muscle element with a typical Hill-type model. Muscle insertion contours within the generic model were mapped onto the subject-specific bone morphology using the non-rigid iterative closest point algorithm.

The surface geometry of each fossa prosthesis type was extracted, and their vertices were set as contact detection points. Condylar contact geometry on the affected side was simplified as an ellipsoid, and the TMJ contact was modeled as a group of contact points to the rotating body. The normal contact force for the TMJ was calculated using a frictionless contact force model for soft materials (Flores and Ambrósio, 2010), with a friction coefficient of 0.001 (Xu et al., 2017).

**FIGURE 4:**
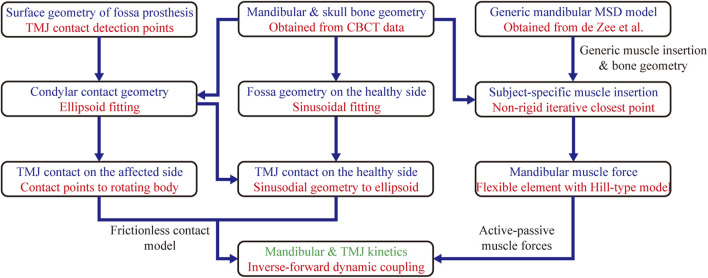
The schematic overview of establishing the subject-specific mandible musculoskeletal model. MSD, Multibody System Dynamics.

Numerical simulations were performed using the inverse-forward dynamic coupling approach (Guo et al., 2022). The 3D mandibular movements were selected as the kinematic inputs to constrain the mandibular bone kinematics. Hyoid location in the intercuspal and maximal opening positions were measured using CBCT, and its movement trajectories were simplified through linear interpolation (Silva and Ambrósio, 2003; Guo et al., 2022). The musculotendon length for each muscle was calculated using the inverse dynamics approach. Motion constraints were removed during forward dynamics estimations. Mandibular muscle forces were estimated via feedback control using their lengths as the target value. A proportional derivative controller was used to calculate the activations for each muscle bundle. The schematic overview of establishing the subject-specific mandible musculoskeletal model is shown in Figure 4.

## 3 Results

**FIGURE 5:**
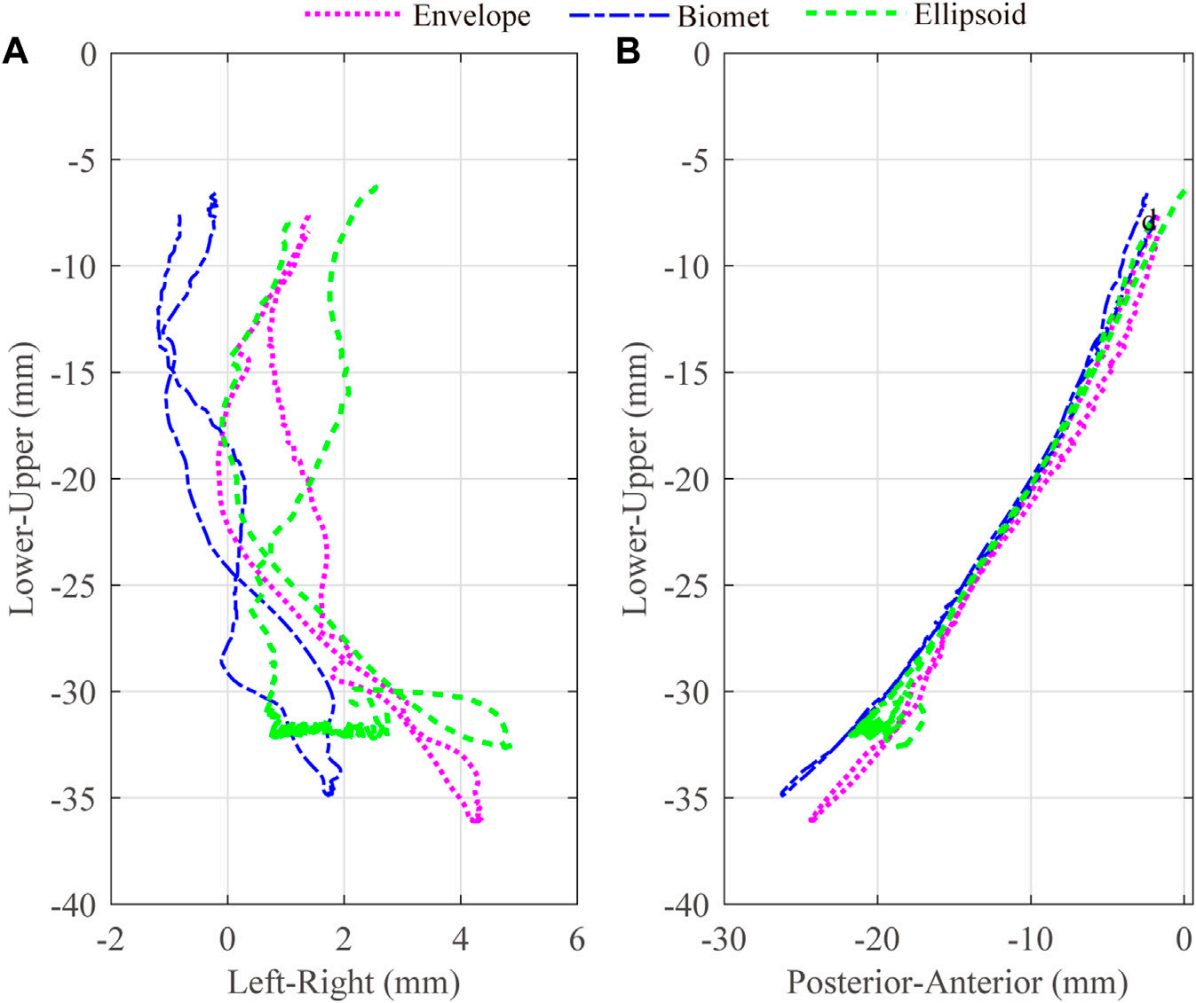
3D trajectories for the lower incisors with different artificial fossae. The axes were separated as two different panes, Left-Right **(A)** and Posterior-Anterior **(B)**.

**FIGURE 6:**
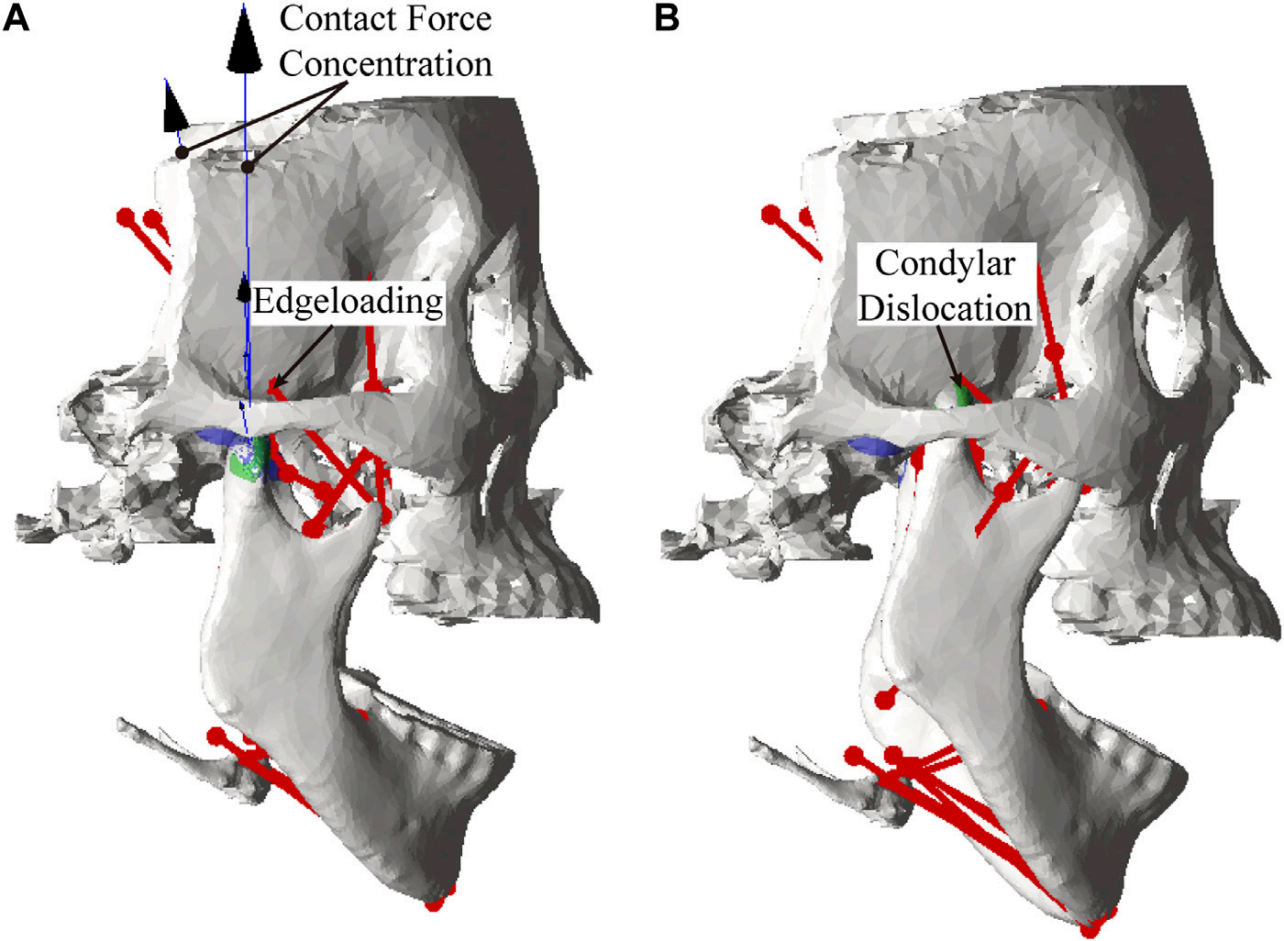
Edgeloading **(A)** and condylar dislocation **(B)** near maximal jaw opening.

Lower incisor movements for different artificial fossa types are shown in Figure 5. The maximum jaw opening magnitudes for the envelope-based, Biomet, and ellipsoidal fossae were 36 mm, 35 mm, and 33 mm, respectively. With an ellipsoidal fossa prosthesis implanted, the condyle of the affected side performed joint dislocation (Figure 6). Condylar ROMs on the affected side were nearly identical for the envelope-based and Biomet prostheses.

**FIGURE 7:**
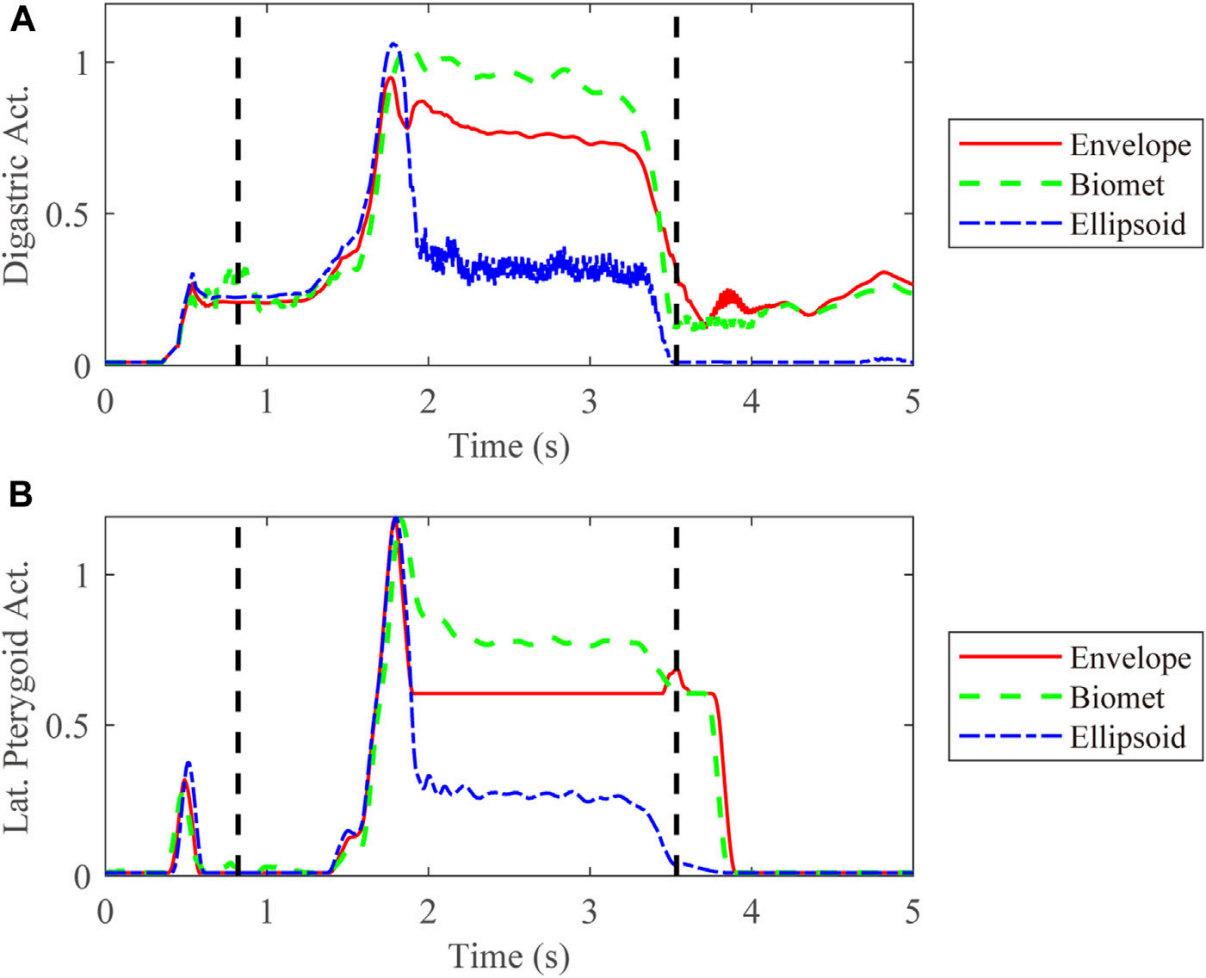
Activation of the digastric **(A)** and lateral pterygoid **(B)** muscles on the affected side. The vertical dotted line demonstrates the time range for jaw opening-closing movements. Act. = Activation. Lat. = lateral.

**FIGURE 8:**
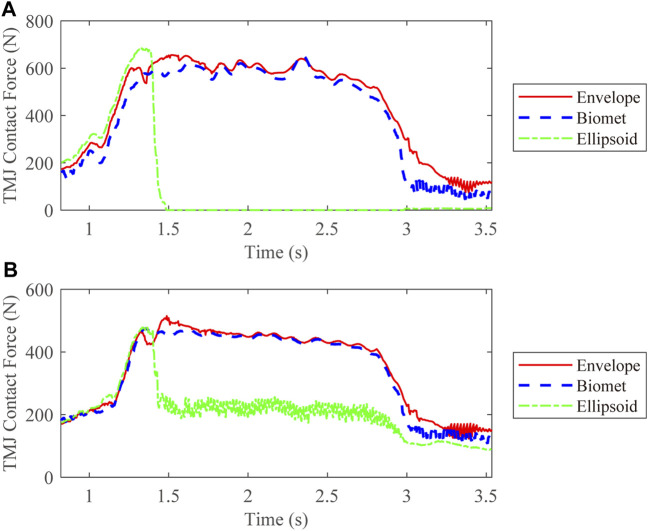
TMJ contact forces for different types of articular fossa types. **(A)** Intact side; **(B)** Affected side.

**FIGURE 9:**
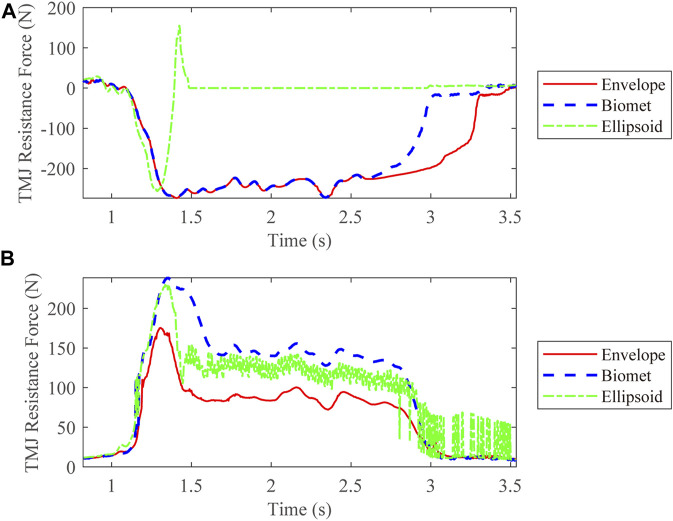
Resistance to forward condylar translation on the intact **(A)** and affected **(B)** sides.

**FIGURE 10:**
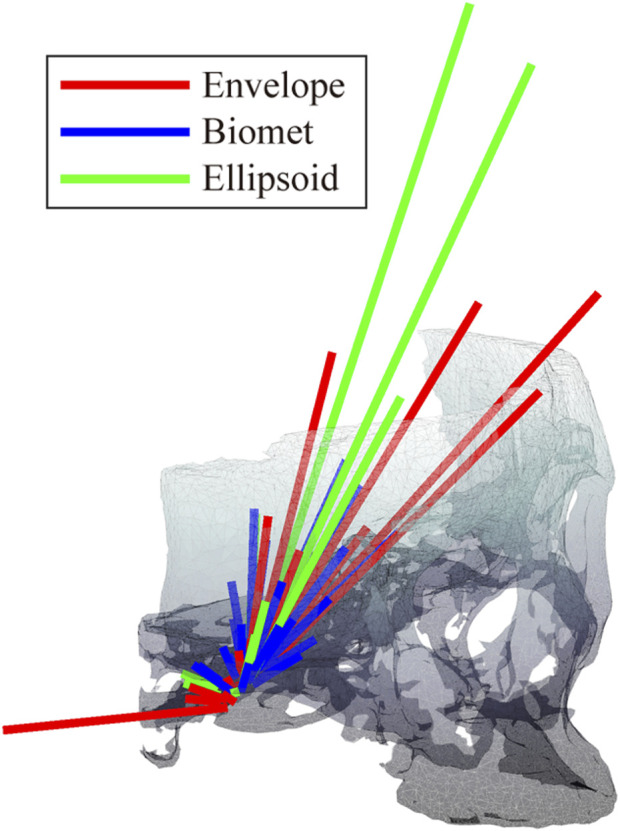
Distribution of TMJ contact forces with the condyles moving to edges of different fossa prostheses.

We also compared the kinetic data during jaw-opening motions. The activation of the digastric and lateral pterygoid muscles during maximal jaw opening was reduced with envelope-based fossa prosthesis compared to Biomet (Figure 7). Bilateral contact forces for the envelope-based and Biomet fossae were similar during and at maximal jaw opening. However, the TMJ with the envelope-based fossa allowed greater normal contact forces than Biomet (Figure 8). When the condyle traveled through the apex, the resistance for forward condylar translation with the envelope-based fossa decreased by 62.4 N (Figure 9). Moreover, the condylar contact force was unevenly distributed for different prostheses, and the maximal contact force for the envelope-based fossa at maximal jaw opening was greater than that for Biomet (Figure 10).

## 4 Discussion

Total joint replacement is commonly used to treat severe degenerative conditions of the TMJ, particularly when conservative treatment has been ineffective. A major goal of TMJ reconstruction is the restoration of normal function. However, current TMJ prostheses that conform to the anatomical shape of TMJ cannot completely restore the physiological condylar kinematics; the geometry of the prostheses reduces the condylar ROM (van Loon et al., 1999; Zheng et al., 2019; Zou et al., 2019). The present study combined the TMJ fossa prosthesis with a functional condylar surface, which was different from the commercially-available TMJ prostheses, allowing physiologically accurate kinematics (Chen et al., 2022b).

The present study was the first to apply the ESCM concept to TMJ fossa prostheses, and a subject-specific mandibular musculoskeletal model was used to simulate mandibular movements, muscle activation, and resistance forces with different prostheses. As a subject-specific model for the human mandibular musculoskeletal system, it had been validated for predicting mandibular trajectories during jaw opening-closing movements in two previous studies, one with seven healthy subjects (Guo et al., 2022), and the other with the patients suffering from oral and maxillofacial tumors (Guo et al., 2023). Calculation precision of the musculoskeletal model would be verified in the further study with patients of TMJ replacement surgery. The kinematic results were compared among the envelope-based, stock Biomet, and ellipsoidal fossa prostheses. The maximum jaw opening magnitude for the stock Biomet fossa prosthesis was 35 mm, which was similar to the long-term outcomes for Biomet following TMJ replacement reported in previous studies (Gonzalez-Perez et al., 2016; Kanatsios et al., 2018). Compared to the Biomet, the envelope-based artificial fossa reduced jaw-opening muscle activation on the affected side and resistance on the intact side. It also increased the maximum jaw opening magnitude while maintaining the condylar ROM and bilateral contact forces. These results suggested that in terms of restoring the natural ROM of TMJ, the ESCM-based TMJ fossa prosthesis did show advantages and potential.

We developed the ellipsoidal fossa prosthesis based on the lower limb joint implants, such as the artificial hip joint (Hernigou et al., 2017). For the ellipsoidal fossa prosthesis design, we only considered condylar rotation and ignored its forward translation. We found that the ellipsoidal fossa limited the postoperative ROM of the TMJ. This indicates the importance of considering all condylar movements while designing prostheses, and reflects the rationale for envelope-based fossa prosthesis.

As showed in the results, there were advantages of the ESCM-based fossa prosthesis. It could not only improve the maximal jaw opening magnitude and condylar ROM, but also increased the efficiency of the jaw-opening process, as shown by the significant decrease in lateral pterygoid activation at maximum jaw opening. These indicated that the ESCM-based artificial fossa successfully replicated the functional anatomy of the mandibular musculoskeletal system. The physiological movement of TMJ could be affected by many factors, such as the posterior slope of articular eminence, the shape and deformation of articular disc, and traction of muscles (Mack, 1989). The articular eminence could provide a stable fulcrum for anterior condyle rotation (Van Eijden et al., 1997), while significant volume of the articular fossa and eminence bone has to be sacrificed for commercial condylar prostheses (Bai et al., 2015). In a study of 165 TMJs reconstructed using the Biomet stock prosthesis, Zhao et al. (2018) found that some patients required significant bone trimming or grafting to adjust the condyle-ramus angle and fossa for stable prosthesis implantation. As a result, the complete structure of articular eminence was damaged. On the other hand, the ESCM-based fossa prosthesis generated by the condylar functional surface data would provide physiological support and guidance for the condylar movement (Chen et al., 2022b; Chen et al., 2023). It may play a combined role of articular eminence, articular disc, capsule, ligament and so on, which could also provide the stable fulcrum for anterior condyle rotation as same as articular eminence. That increased the moment arms of the jaw-opening muscles (Spencer, 1998) and so could explain the significant decrease in digastric activation at the maximal jaw opening. Moreover, the guidance of ESCM-based fossa prosthesis for the condyle to slide forward could explain the significant decrease in lateral pterygoid activation at maximum jaw opening.

This study also had some limitations. First, the data used were obtained from a single subject. Studies with larger sample sizes will be required in the future. In addition, the subject of this study had no history of TMJ diseases, while total TMJ replacement is a biomechanical treatment option for patients with end-stage TMJ diseases (Sidebottom, 2008). For the patients whose articular fossa is damaged by the tumor and the condyle is intact and the condylar movement is normal, their envelope surfaces of condylar movement can still be obtained by the method used in this study. Moreover, in the case where the unilateral condyle is damaged but the patient could still perform normal mandibular movements, the mirroring of the intact side could be applied using the same method. This method could not be applied to those patients who were unable to perform normal mandibular movements, such as, patients with TMJ ankylosis. A method for predicting the shape of ESCM based on the facial morphology had been proposed in the previous study (Chen et al., 2023). Although further research was needed, it may be helpful in future ESCM data collection of patients with TMJ diseases whose normal mandibular movements could not be performed. Second, the complex TMJ morphology and loading patterns were simplified for our musculoskeletal models. TMJ cartilage and articular disk of the intact side were not modelled, which may have influenced the contact mechanics of the intact side. Besides, given that the mandibular kinematics would change for different other positions, more mastication loading conditions other than the maximum intercuspal position should be considered in the further study. Third, the condylar geometry on the affected side was simplified as an ellipsoid. An articular fossa prosthesis should be matched with a suitable condylar prosthesis based on the patient-specific functional anatomy. ESCM-based fossa prostheses still require some improvements. For example, the peak contact force for the envelop-based fossa was greater than that for Biomet at maximum jaw opening. This may have been because of the uneven distribution of contact forces, resulting from the bistable shape of the envelope-based fossa prosthesis, which could be influenced by the articular eminence morphology (Huang et al., 2021; Chen et al., 2022b). Compared with the envelope-based artificial fossa, the surface geometry of the Biomet fossa was flatter, making the contact force distribution more even. The uneven geometry of the envelope-based fossa prosthesis reduced the contact area with the condyle, resulting in an increased contact force. This would increase the potential for component wear, material failure, and TMJ dislocation (Kent et al., 1986; Giannakopoulos et al., 2012). Moreover, an anterior stop of the ESCM-based fossa prosthesis may be needed to avoid TMJ luxation, which will be considered in the further study. Despite these limitations, the study offers a novel perspective for TMJ fossa prostheses design. The customized envelope-based fossa prosthesis described in this study may allow the optimization of TMJ fossa prosthesis design.

## 5 Conclusion

A customized TMJ fossa prosthesis was successfully developed using the ESCM concept. Our study of musculoskeletal multibody modeling has highlighted its advantages and potential. The artificial fossa design successfully achieved a wider condylar ROM. It also reduced the activation of jaw opening muscles on the affected side and resistance on the intact side. This study showed that an ESCM-based approach may be useful for optimizing TMJ fossa prostheses design.

## Data Availability

The raw data supporting the conclusion of this article will be made available by the authors, without undue reservation.
